# Electrochemical in-biosensing computing

**DOI:** 10.1093/nsr/nwag290

**Published:** 2026-05-18

**Authors:** Cheng Yuan, Ao Xiao, Shuang Wu, Xing-Shi Liu, Jing-Juan Xu, Hong-Yuan Chen, Wei-Wei Zhao

**Affiliations:** State Key Laboratory of Analytical Chemistry for Life Science, School of Chemistry, Nanjing University, Nanjing 210023, China; State Key Laboratory of Analytical Chemistry for Life Science, School of Chemistry, Nanjing University, Nanjing 210023, China; State Key Laboratory of Analytical Chemistry for Life Science, School of Chemistry, Nanjing University, Nanjing 210023, China; State Key Laboratory of Analytical Chemistry for Life Science, School of Chemistry, Nanjing University, Nanjing 210023, China; State Key Laboratory of Analytical Chemistry for Life Science, School of Chemistry, Nanjing University, Nanjing 210023, China; State Key Laboratory of Analytical Chemistry for Life Science, School of Chemistry, Nanjing University, Nanjing 210023, China; State Key Laboratory of Analytical Chemistry for Life Science, School of Chemistry, Nanjing University, Nanjing 210023, China

**Keywords:** electrochemical biosensing, in-sensor, computing, neuromorphic, AI

## Abstract

Artificial intelligence (AI)-aided electrochemical biosensing is becoming integral parts in numerous scenarios. However, existing systems generally perform algorithms in external signal processing units. The necessity of analog-to-digital conversion and data transfer results in high complexity, low working efficiency and concern of privacy. In-sensor computing has made great progress in perceiving and processing physical signals, which, nevertheless, faces inherent restriction in biochemical scenarios due to the lack of aqueous compatibility and the necessity of an array. Here, we realized neuromorphic electrochemical in-biosensing computing using just a single photoelectrochemical transistor, which can itself not only perform multi-target biosensing but also constitute a single-layer algorithmic classifier. It is based on a rationally designed multi-gate photoelectrochemical transistor, whose architecture and synaptic memory enable built-in vector-matrix multiplication and light-tunable responsivity. The proof-of-concept is demonstrated by simultaneous sensing and classification of biomarker microRNA fingerprints in real biological samples, which opens the possibilities for next-generation AI-driven electrochemical biosensing with edge computing ability.

## Teaser

This study realizes the in-sensor computing in the field of electrochemical biosensing using just a single neuromorphic organic photoelectrochemical transistor for the first time.

## INTRODUCTION

Conventional electrochemical biosensing typically generates numerical values for target concentrations, leaving interpretation of such values up to the users [1,2]. Driven by the improved robustness and minimized information loss, recent artificial intelligence (AI)-aided electrochemical biosensing has achieved huge success [3,4], especially in classification task [5–8], which could provide more interpretable outputs to identify specific physiological and pathological states. However, existing systems generally follow a workflow of ‘biosensing, analog-to-digital conversion, data transfer, and decision-making’, i.e. the AI algorithms are implemented in external signal processing units, leading to higher complexity, lower efficiency and concern of data security [9,10]. Recently, van de Burgt *et al.* [9] and Gao *et al.* [10] reported the integration of sensing and neuromorphic computing on the chips, which, nevertheless, still consists of physically separated sensing and computing modules. Empowering electrochemical biosensing with capabilities of analog signal processing and real-time computing capability to directly yield decisive results—by *in-situ* deployment of AI algorithms—remains a pivotal challenge [11–16].

By integrating sensors with neurosynaptic devices, in-sensor computing capable of *in-situ* computing within sensors has been demonstrated powerful in simultaneous sensing and processing of physical signals, e.g. electricity, light and pressure [17–23]. Despite ongoing technical advancements [11–16], critical issues remain: (i) Existing solid-state systems can only perceive limited types of physical signals. (ii) Deploying the in-sensor neural network necessitates an interconnected device array for the vector-matrix multiplication (VMM) based on Kirchhoff’s laws. To apply in-sensor computing in biochemical sensing still faces many inherent restrictions, e.g. lack of aqueous compatibility, device-to-device variability and crosstalk noise [24,25]. To date, electrochemical in-biosensing computing on a single device remains a formidable challenge.

Aqueous neuromorphic engineering with biosensing capability is burgeoning as it holds great promise in many sectors [26–31]. Among them, we fused electrochemical transistor [32–34] and photoelectrochemistry [35–38] to propose organic photoelectrochemical transistor (OPECT) [39]. In its operation, dual electrolyte- and photo-gating allows the rich light-matter-bio interplay in aqueous environments and thus close connection with diverse biological events [40]. Moreover, due to its synaptic resemblance, it has later been shown versatile in developing various neurosynaptic devices with unknown possibilities [41–48]. For example, Inal *et al.* developed a p-type OPECT synapse exhibiting broad-band light detection spanning the visible to near-infrared range [45]. Huang *et al.* recently developed an OPECT array capable of sensing near-infrared light and performing convolutional computing [46]. Significantly, in an earlier work, we have observed unique memory characteristics and especially the light-tunable biochemical responsivity (*R*) in the aqueous device [47]. Such interesting properties constitute the core of VMM and thus open the possibility to deploy electrochemical in-biosensing computing.

To address this knowledge gap, we used a single synaptic OPECT to realize electrochemical in-biosensing computing for the first time. The concept was exemplified by a rationally designed three-gate OPECT, which itself can not only execute multi-target biosensing but also artificial neural network (ANN), i.e. a single-layer classifier. Principally, its operation was based on (i) utilizing its synaptic memory to perform accumulation and to afford built-in VMM between concentrations and *R* matrix without the need of a transistor array; (ii) the linear relationship between logarithmic value of light intensity and *R* values enabling deterministic weight updating via adjusting light intensity. Using this device, a complete electrochemical in-biosensing computing system was constructed, which can sense multiple targets, and based on this, generate reliable algorithmic decision-making. The proof-of-concept classification of biomarker microRNA fingerprints were realized in real biological samples, with superior robustness, sensitivity, and accuracy.

## RESULTS AND DISCUSSION

### Design and rationale

**Figure 1.**
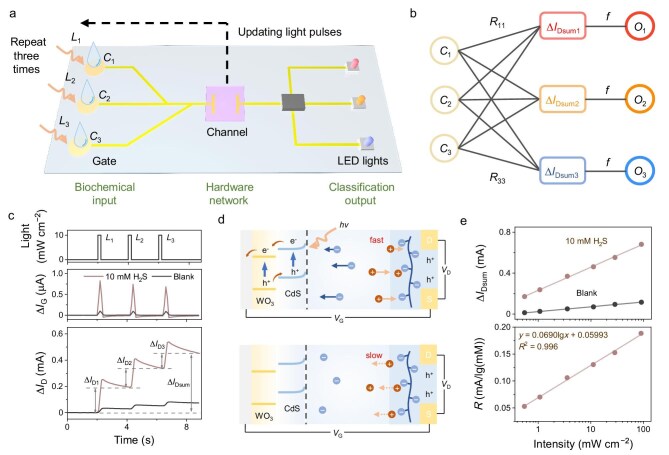
(a) Schematics of the architecture of the device linked with LED lights. (b) Schematics of the in-biosensing ANN classifier. (c) The ∆*I*_G_ and ∆*I*_D_ responses of the device before and after the treatment of 10 mM H_2_S upon three light pulses on the three photogates. (d) The mechanism of memory from the perspective of electron transferring and ion migration upon and after light illumination. (e) The ∆*I*_Dsum_ responses of the device before and after the treatment of H_2_S, and corresponding *R* values upon three light pulses with different light intensities on the three photogates.

Figure 1a schematically illustrates the architecture of the synaptic OPECT, which was constructed by the poly(3,4-ethylenedioxythiophene) poly(styrenesulfonate) (PEDOT:PSS) channel gated by three parallel Cd^2+^/WO_3_ photoelectrodes (see Figs S1–S4 for characterization) in the phosphate buffered saline (PBS) electrolyte. Upon three subsequent light pulses with individual intensities (denoted as (*L*_1_, *L*_2_, *L*_3_)), the three photogates could respectively perceive different biochemical inputs (concentration of the targets, denoted as (*C*_1_, *C*_2_, *C*_3_)) and thus generate specific photovoltages to co-modulate the channel conductivity. Such a design permitted not only VMM operation for forward propagation but also tunable *R* matrix for backward propagation, forming the basis for the proposed in-biosensing ANN. Incidentally, if such a task was operated on a conventional crossbar array, many transistors with high complexity and energy consumption would be necessary (Fig. S5).

Specifically, upon light illumination, each photogate conducted a channel current variation of ∆*I*_D_ = *R_n_* × (lg*C*_*n*_ + *b*), where *n* denoted the photogate index (*n* = 1, 2, 3), *b* was a constant and *R* was the biochemical responsivity. Significantly, due to the memory effect of *I*_D_, the OPECT itself could perform the direct accumulation, producing a summed current variation of ∆*I*_Dsum_ = Σ*R_n_* × (lg*C*_*n*_ + *b*). Such a process was then repeated for three times using all nine *R* values to perform the VMM and obtain the complete output (∆*I*_Dsum1_, ∆*I*_Dsum2_, ∆*I*_Dsum3_) (Fig. S6). On the other hand, each *R_n_* could be individually tuned by the light intensity *L_n_*, making possible the updating of *R_n_* matrix for training. To reduce the influence of nonlinearity, *R* was calculated using averaged value of ∆*I*_D1_, ∆*I*_D2_ and ∆*I*_D3_: *R* = (∆*I*_Dsum_(with biochemical inputs) − ∆*I*_Dsum_(without biochemical inputs))/3lg*C* upon three subsequent light illumination on the three photogates. Based on such properties, a 3 × 3 in-biosensing single-layer classifier could be deployed (Fig. 1b), which consisted of an input layer of concentrations (*C*_1_, *C*_2_, *C*_3_), synaptic weights encoded in a *R_n_* matrix (*R*_11_–*R*_33_), the corresponding *L* matrix(*L*_11_–*L*_33_) to control *R*, ∆*I*_Dsum_ results (∆*I*_Dsum1_, ∆*I*_Dsum2_, ∆*I*_Dsum3_), activation function (*f*) and an output layer (*O*_1_, *O*_2_, *O*_3_) (see Methods for detailed setting). Compared with conventional electrochemical approaches relying on extra data analysis after biosensing, the device could *in-situ* process the multiple analog signals and then directly display the results through the ∆*I*_D_-controlled LED lights.

To experimentally validate its feasibility for in-biosensing ANN, H_2_S was chosen as a model analyte, which can react with Cd^2+^ and produce CdS/WO_3_ type-II heterojunction, leading to enhanced photoresponses (Figs S7–S14). Upon individual light pulses on the three photogates, the OPECT could produce three respective gate currents (∆*I*_G_). Due to its intrinsic synaptic memory (Fig. S15), the corresponding ∆*I*_D_ exhibited an accumulated ∆*I*_Dsum_, which was equaled to the sum of all the single ∆*I*_D*n*_ (Fig. 1c). Both the pulse width and the interval were optimized (Fig. S16). Considering that each ∆*I*_D_ was proportionally correlated to the respective H_2_S concentration in the linearity range (Fig. S17), the VMM operation could be performed. The mechanisms were illustrated (Fig. 1d). Upon light illumination, the photo-induced charge transfer would produce positive photovoltage, leading to the fast injection of cations into the channel. During the intervals of light illumination, the slow migration of the cations out of the channel resulted in the memory of ∆*I*_D_. On the other hand, the light-tunable *R* was investigated. Compared to the pristine ∆*I*_Dsum_, the ones after H_2_S treatment were enhanced (Fig. 1e). The mechanism was illustrated from the perspective of transfer characteristic and ion migration (Figs S18 and S19). With increased *L*, the ∆*I*_Dsum_ and corresponding *R* values (calculated by ∆*I*_Dsum_/lg*C*) were increased and exhibited good linearity with the logarithm of *L*, which could be used for deterministic synaptic weights updating. Such a sublinear dependence could be attributed to the different rate-limiting step at low and high light intensity. The semiconductor performance was determined by the hole transfer to the surface at low light intensity while it was limited by hole transfer to the electrolyte at high light intensity. With increased light intensity, the band bending became flattened, limiting the hole transferring to the electrolyte so that the photo-induced voltage gradually got saturated [49].

### Training and classification

**Figure 2.**
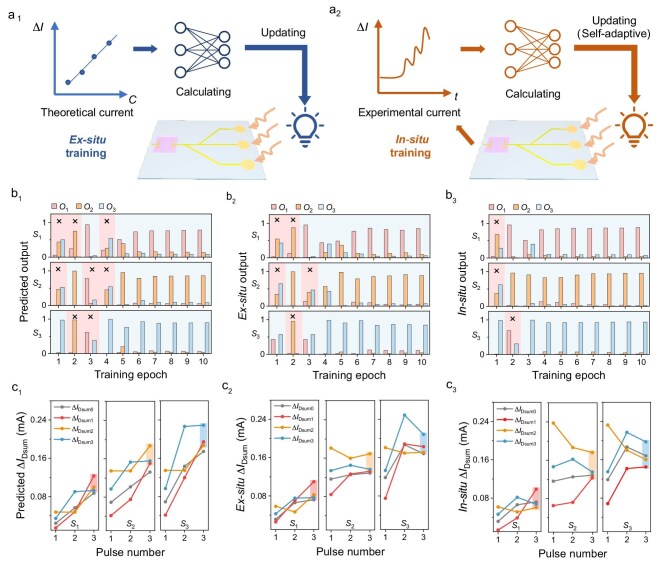
(a) Schematic of (a_1_) *ex-situ* training and (a_2_) *in-situ* training. (b) The outputs of the ANN classifier obtained by (b_1_) theoretical prediction, (b_2_) *ex-situ* training and (b_3_) *in-situ* training when inputting *S*_1_–*S*_3_. (c) The eventual ∆*I*_Dsum_ (subtracting the blank ∆*I*_Dsum_) obtained by (c_1_) theoretical prediction, (c_2_) *ex-situ* training and (c_3_) *in-situ* training after 10 training epochs when inputting *S*_1_–*S*_3_.

*Ex-situ* and *in-situ* training are two typical training modes of in-sensor ANNs [50]. In *ex-situ* training, weights are calculated based on theoretically fitted functions and then programmed into the system, which is easily influenced by random errors, e.g. departure from linearity and batch-to-batch variation (Fig. 2a_1_). By contrast, *in-situ* training, where weights are self-adaptively updated according to the real-time experimental data, can tolerate more non-idealities (Fig. 2a_2_). Here, to investigate the classification capability of our device, we explore a typical linear separation task with concentration combinations of H_2_S as the model samples (denoted as *S*_1_ = (100 μM, 100 μM, 100 μM), *S*_2_ = (1 mM, 100 μM, 100 μM) and *S*_3_ = (1 mM, 1 mM, 100 μM)). For classification, a random sample was inputted, with the three concentrations of H_2_S respectively reacted with the three photogates, generating ∆*I*_Dsum1_, ∆*I*_Dsum2_, and ∆*I*_Dsum3_. If the index *n* of the highest ∆*I*_Dsum_ was equaled to that of the inputted *S_n_*, the result was right. During training, the *R* matrix as well as the *L* matrix were repeatedly adjusted, until all the samples were correctly classified.

The predicted results showed that after 5 epochs, the OPECT could successfully classify all the samples with 8 mistakes in the initial 4 epochs (Fig. 2b_1_). However, by *ex-situ* training, after 4 epochs, the device could classify all the samples with 5 mistakes in the initial 3 epochs, which could be attributed to the difference between theoretical and experimental ∆*I*_Dsum_ (Fig. 2b_2_). Significantly, by *in-situ* training, after only 3 epochs, the device could classify all the samples with 3 mistakes in the initial 2 epochs, indicating the much-improved training efficiency (Fig. 2b_3_). The variation of synaptic weights and decreased Loss values also validated the successful training (Figs S20–S22). Before training, the device could only produce three identical ∆*I*_Dsum_ (denoted as ∆*I*_Dsum0_) due to the same *L*. After training, it could produce three different ∆*I*_Dsum1_, ∆*I*_Dsum2_, and ∆*I*_Dsum3_ due to the weight updating (Fig. 2c_1_–c_3_). Specifically, *S*_1_, *S*_2_, *S*_3_ would result in the highest ∆*I*_Dsum1_, ∆*I*_Dsum2_, ∆*I*_Dsum3_, respectively, indicating the ∆*I*_Dsum_-based direct classification without the necessity of analog-to-digital conversion. Note that *in-situ* training resulted in larger differences between ∆*I*_Dsum1_, ∆*I*_Dsum2_ and ∆*I*_Dsum3_, indicating its superior classification capacity.

**Figure 3.**
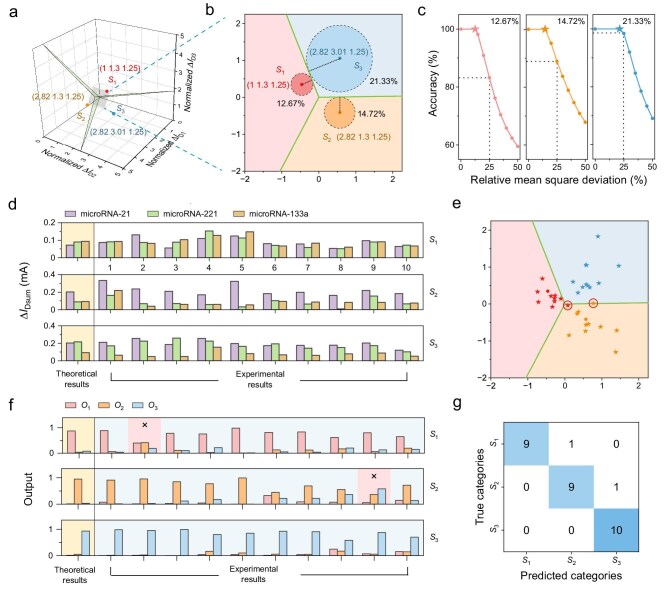
(a) Three-dimensional graphical representation of the decision surfaces and samples. (b) The projection of the decision surfaces and sample points on the normal plane. (c) Theoretical accuracy with increased relative mean square deviation. (d) The ∆*I*_Dsum_ (subtracting the blank ∆*I*_Dsum_) upon three light pulses on each individual photogate of 30 random devices for classification. (e) The projection of the ∆*I*_Dsum_ on the normal plane. (f) The corresponding outputs of 30 devices for classification. (g) The corresponding confusion matrix.

The robustness was then theoretically and experimentally investigated. Based on the weight matrix obtained from the *in-situ* training, the functions of decision surfaces and normalized coordinates of the three samples were calculated (Fig. 3a and Note S1). As shown, the surfaces divided the whole space into three parts, and the three fingerprints were located in each one of them. To study the relationship between the device-to-device variation and classification accuracy, the surfaces and three sample points were projected into the normal plane of the surfaces (grey square). In the normal plane, the distance between samples and the closest boundary represented the highest mean square deviation that the device could tolerate for 100% accuracy (Fig. 3b). The results demonstrated that, for the accurate classification of *S*_1_, *S*_2_ and *S*_3_, the device could tolerate maximum relative mean square deviations of 12.67%, 14.72% and 21.33%, respectively, which are much higher than the common errors in electrochemical biosensors. Further increase of the error would result in decreased accuracy. However, the accuracy could still maintain higher than 80% when the deviation increased to 25%, indicating its excellent robustness (Fig. 3c).

Experimentally, without any intentional selection, 30 devices were randomly used for the classification of *S*_1_–*S*_3_. Upon three light pulses on each individual photogate, the ∆*I*_Dsum_ was measured to reveal the differences between the device responses and the theoretical results (Fig. 3d). Compared with the theoretical results, the experimental ∆*I*_Dsum_ of three photogates exhibited randomly enhanced or decreased values, indicating the existence of device-to-device variation. The data was then used for predicting the accuracy by projecting all the ∆*I*_Dsum_ of the 30 devices into the normal plane of decision surfaces (Fig. 3e). As shown, most of the points were located within the correct parts, with only 2 mistakes, i.e. one red point belonging to the *S*_1_ was separated into the orange *S*_2_ part and one orange point belonging to the *S*_2_ was separated into the blue *S*_3_ part. On the other hand, despite the device-to-device variation, the experimental outputs revealed that the device could successfully classify most of the fingerprints with only 2 mistakes, which was totally consistent with the prediction (Fig. 3f). The corresponding confusion matrix demonstrated the overall accuracy of 93.33%, confirming the excellent robustness (Fig. 3g).

### MicroRNA fingerprints classification

In biological fluids, the levels of multiple biomarkers are often associated with specific physiological and pathological processes. Simultaneous measurement of them is particularly valuable because of their relevant quantitative information, which has important diagnostic and prognostic implications [51]. For example, microRNA fingerprints have been established as key biomarkers for diagnosis of various diseases [52]. Here, a proof of concept was demonstrated by classification of three microRNA fingerprints in real biological samples. Specifically, *F*_1_ used the cell lysate of MCF-10A, which expressed low-level microRNA-21, microRNA-221 and microRNA-133a. *F*_2_ used the cell lysate of MCF-7, which expressed high-level microRNA-21 and low-level microRNA-221 and microRNA-133a. *F*_3_ used the cell lysate of HepG-2, which expressed high-level microRNA-21 and microRNA-221 and low-level microRNA-133a. With the assistance of nucleic acid strategies (Figs S23 and S24), each of the three photogates individually responds to one of specific microRNA, which exhibited good linearity and selectivity (Figs S25–S28).

**Figure 4.**
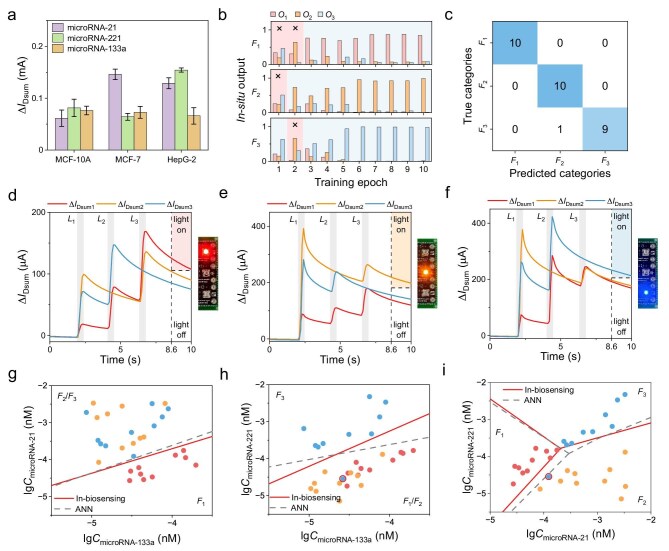
(a) The ∆*I*_Dsum_ upon three light pulses on each individual photogate with the treatment of cell lysates from MCF-10A, MCF-7 and HepG-2. The standard deviations are from 3 different samples. (b) The *in-situ* training outputs of every training epoch when inputting *F*_1_–*F*_3_. (c) The confusion matrix testing 30 cell lysate samples. (d–f) The ∆*I*_Dsum_ and corresponding turning on or off of the LED lights when inputting (d) *F*_1_, (e) *F*_2_, and (f) *F*_3_. (g–i) The decision boundaries obtained from this method and conventional ANN method when analyzing the (g) microRNA-133a and microRNA-21, (h) microRNA-133a and microRNA-221 as well as (i) microRNA-21 and microRNA-221.

In the case of *F*_1_, all the photogates induced similar low ∆*I*_Dsum_ responses (Fig. 4a). In the case of *F*_2_, the photogate corresponding to microRNA-21 generated substantially higher response. In the case of *F*_3_, the photogates corresponding to microRNA-21 and microRNA-221 both generated higher responses. Such results were well consistent with the expression levels in three cell lines. *F*_1_–*F*_3_ were then used for *in-situ* training. After three epochs, the device could successfully classify all the fingerprints (Fig. 4b). The variation of synaptic matrix and decreased Loss values also proved the successful training (Figs S29–S31). Analyzed by the aforementioned theoretical model, the device could tolerate large relative mean square deviations up to 22.25%, 24.06% and 10.31% for 100% accurate classification of *F*_1_, *F*_2_ and *F*_3_, respectively (Fig. S32). Further experimental validation demonstrated that only 1 mistake was made among 30 cell lysate samples, confirming its excellent robustness (Fig. 4c and Figs S33–S35).

To rapidly and directly present the classification results, the OPECT was linked with a delay circuit and a threshold circuit to control the LED lights (Figs S36 and S37). Specifically, ∆*I*_Dsum1_–∆*I*_Dsum3_ were respectively measured and compared with the thresholds in the threshold circuit to determine which light should be turned on after only 8.6 s. In the case of *F*_1_, the device would produce highest ∆*I*_Dsum1_ over the threshold after calculation, resulting in the turning on of the red light (Fig. 4d). On the other hand, in the case of *F*_2_ (Fig. 4e) and *F*_3_ (Fig. 4f), the highest ∆*I*_Dsum2_ and ∆*I*_Dsum3_ would lead to the turning on of orange and blue light, respectively. Such an operation permitted fast and direct demonstration of the results.

In the machine learning-aided bioanalysis, the decision boundaries were commonly used to determine the concentration ranges of biomarkers corresponding to normal and abnormal status [53]. Conventionally, to obtain the decision boundaries, the samples were initially detected by sensors, the generated data was then calculated into concentrations via as-fitted linearity functions. Subsequently, the concentration information must be transferred and analyzed by algorithm (Fig. S38). By contrast, the device could produce decision boundaries without the necessity of calculating concentrations and their transferring. Specifically, after detecting the sample, the raw current data was fed back for weight updating. After training, only the *R* matrix was extracted to produce the decision boundaries. Here, the decision boundaries of the conventional method and in-biosensing ANN were compared. When analyzing the microRNA-133a and microRNA-21, both the decision boundary of this method and conventional ANN method could successfully separate the *F*_1_ with *F*_2_/*F*_3_ (Fig. 4g). When analyzing the microRNA-133a and microRNA-221, both the decision boundaries successfully separated the *F*_3_ with *F*_1_/*F*_2_ with only 1 mistake (Fig. 4h). When analyzing the microRNA-21 and microRNA-221, both the decision boundaries successfully separated the *F*_1_, *F*_2_ and *F*_3_ with only 1 mistake (Fig. 4i). The decision boundaries of this method were very similar to that of the conventional method (see Note S2 for their calculation and functions). Incidentally, this method could also be used for the calculation of specific concentrations (Fig. S39). This strategy could be extended to a bigger neural network and other targets (Figs S40 and S41). This strategy could be further improved from the perspective of computing speed by optimizing the geometric structure of the device and the introduction of parallel computing. Moreover, the ANN in this article is single-layer. Expanding the ANN into a multi-layer one by utilizing more devices can extend the application for more complex nonlinearly separable problems (Fig. S42).

## CONCLUSION

In conclusion, we realized electrochemical in-biosensing computing with only a single synaptic OPECT, which itself can simultaneously perform multi-target biosensing and ANN classification. Due to its rationally designed architecture and intrinsic synaptic memory, the device could achieve built-in VMM operation and light-tunable weights for *in-situ* ANN training. In a proof-of-concept application, it was utilized for microRNA fingerprints classification without manual data analysis, analog-to-digital conversion, and data transfer to external computing units. As compared to conventional approaches based on device array, this single-transistor strategy could reduce complexity and energy consumption. This strategy can also be generalized to other biological applications by changing the biosensing events. Further optimization of the geometric structure and the introduction of parallel computing can improve the operation speed. Moreover, expanding the ANN to multilayers can permit application for nonlinear problems. Overall, by deploying algorithm into a single synaptic OPECT, this is a first report of electrochemical biosensing with real edge-computing ability, which is expected to offer a transformative paradigm to simpler, securer, cheaper, and more robust next-generation AI-driven electrochemical biosensing.

## METHODS

### Fabrication setup

To fabricate the Cd^2+^/WO_3_ gate, 0.0618 g Na_2_(WO_4_)_2_•2H_2_O, 2.5 mL of 3 M HCl and 0.0586 g (NH_4_)_2_C_2_O_4_•6H_2_O (from Sigma-Aldrich Reagent Company; China) were dissolved in 15 mL deionized water and then transferred into a Teflon container. The FTO substrates were immersed in the solution and kept at 140°C for 3 h. After washing by deionized water, the electrodes were calcined in a muffle furnace at 450°C for 2 h and immersed in 1 mM CdCl_2_ (from Sigma-Aldrich Reagent Company; China) solution. To fabricate the channel, 0.1 mL PEDOT:PSS dispersion containing 5% v/v dimethyl sulfoxide was dropped onto the substrate, spin-coated (4000 r/min 30 s) and annealed at 180°C for 1 h. To reduce the variability, photogates with similar photocurrents and channels with similar conductivity were initially selected.

### Experimental setup

For microRNA sensing, carboxylated MBs (1 mg/mL) was activated by carbodiimide hydrochloride (2.5 mg/mL) and *N-*hydroxysuccinimide (2.5 mg/mL) for 15 min. Then, 100 μL of H1 (1 μM) was added and reacted for 9 h at 37°C and then blocked by adding 100 μL BSA aqueous solution (1 mg/mL) for 2 h. Next, 100 μL of target microRNA, H2 (1 μM), ALP-H3 (1 μM) and ALP-H4 (1 μM) were added and reacted for 4 h at the same condition. After magnetic separation, the MBs were added to 1 mL TP aqueous solution (1 mM) and reacted for 2 h. The MBs were separated and washed after every step. All the cells were seeded in plates and in a 5% CO_2_ environment at 37°C. The electrical signals were recorded in 0.1 M PBS and the channel voltage was kept as 0.2 V during testing.

### Training setup of the ANN classifier

Before training, the *L* values in the initial 3×3 *L* matrix was identical. During forward propagation, the three *L* values in the list of the 3×3 *L* matrix was applied on the device to obtain a ∆*I*_Dsum_. Such process was repeated for 3 times to perform the complete VMM operation between input vector (lg*C*_1_, lg*C*_2_, lg*C*_3_) and *R_n_* matrix to obtain the ∆*I*_Dsum1_−∆*I*_Dsum3_. Subsequently, on the computer, those current values were then activated by SoftMax function for normalization. During backward propagation, the cross entropy was selected as Loss function to evaluate the difference between the experimental outputs and the ideal ones. A new *R_n_* matrix was calculated via gradient descent. Specifically, for *ex-situ* training, the functions in Fig. S20 were used to calculate the theoretically predicted ∆*I*_Dsum1_−∆*I*_Dsum3_ and thus the *R* matrix of every epoch. For *in-situ* training, the experimental ∆*I*_Dsum1_−∆*I*_Dsum3_ of every epoch were used to calculate the *R* matrix. Once a new *R* matrix is obtained, a new *L* matrix could be calculated according to the fitted functions in Fig. 2c, which was used for the forward propagation in the next epoch.

## Supplementary Material

nwag290_Supplemental_File
