# Supplementary material for: Electrochemical in-biosensing computing
Source: Natl Sci Rev. 2026 May 18;13(12):nwag290. doi: 10.1093/nsr/nwag290 (PMC13321122; doi:10.1093/nsr/nwag290)
Supplement: nwag290_Supplemental_File [file nwag290_supplemental_file.pdf]

Supporting Information for

Electrochemical In-Biosensing Computing

Cheng Yuan, Ao Xiao, Shuang Wu, Xing-Shi Liu, Jing-Juan Xu, Hong-Yuan Chen, Wei-Wei Zhao*

State Key Laboratory of Analytical Chemistry for Life Science, School of Chemistry, Nanjing University, Nanjing 210023, China
*Corresponding author. E-mail: zww@nju.edu.cn

Figure S1. The SEM image of WO_3.
Figure S2. The EDS mapping images of WO_3.
Figure S3. The XRD curve of WO_3/FTO.
Figure S4. Fabrication step and image of the device.
Figure S5. Schematic of the in-biosensing 3×3 neural network.
Figure S6. Schematic illustration of the in-sensor VMM process.
Figure S7. The SEM image of CdS/WO_3.
Figure S8. The EDS mapping images of CdS/WO_3.
Figure S9. The UV-vis spectra of CdS/WO_3 and WO_3.
Figure S10. The XPS spectra of CdS/WO_3 and WO_3.
Figure S11. Transient photocurrent responses of CdS/WO_3 and WO_3.
Figure S12. The electron transfer routes upon light illumination.
Figure S13. The operation stability of CdS/WO_3 and WO_3.
Figure S14. The storage stability of CdS/WO_3 and WO_3.
Figure S15. The ΔI_D and OCP upon light pulse.
Figure S16. Optimization of the pulse width and interval.
Figure S17. TheΔI_{Dsum} responses with the treatment of H_2S with variable concentrations.
Figure S18. The transfer characteristic curves before and after the treatment of H_2S upon light illumination with different intensities.
Figure S19. The ion migration of WO_3 and CdS/WO_3 upon light illumination.
Figure S20. The predicted variation of *L* matrix.
Figure S21. The variation of *L* matrix during *in-situ* training.
Figure S22. The variation of Loss during training.
Note S1. The calculation of functions of decision surfaces and coordinates of fingerprints.
Figure S23. Nucleic acid strategy for microRNA biosensing.
Figure S24. PAGE analysis of nucleic acid strategy.
Figure S25. Reponses before and after the treatment of micro RNA.
Figure S26. The calibration curves of microRNA detection.
Figure S27. The selectivity of microRNA detection.
Figure S28. The process of microRNA fingerprint sensing.
Figure S29. The predicted variation of *L* matrix towards classification of real biological samples.
Figure S30. The variation of *L* matrix during *in-situ* training towards classification of real biological samples.
Figure S31. The variation of Loss during training towards classification of real biological samples.
Figure S32. The projection of the decision surfaces and fingerprints of real biological samples on the normal plane.
Figure S33. The ΔI_{Dsum} upon three light pulses on each individual photogate of 30 random samples.
Figure S34. The projection of the corresponding ΔI_{Dsum} on the normal plane.
Figure S35. The outputs of classifying 30 samples.
Figure S36. Schematic illustration of the device controlling LED lights.
Figure S37. Image of the delay and the threshold circuits.
Figure S38. Schematic illustration of decision boundary acquisition using conventional method and in-biosensing ANN.
Note S2. Functions of decision boundaries and coordinates of fingerprints.
Figure S39. The comparation of microRNA concentrations calculated by this method and linearity functions.
Figure S40. Schematic of an in-sensor N×M ANN.
Figure S41. The in-sensor ANN with the input of protein.
Figure S42. Schematic illustration of the in-biosensing multi-layer ANN.
Table S1. DNA sequences used for microRNA detection.

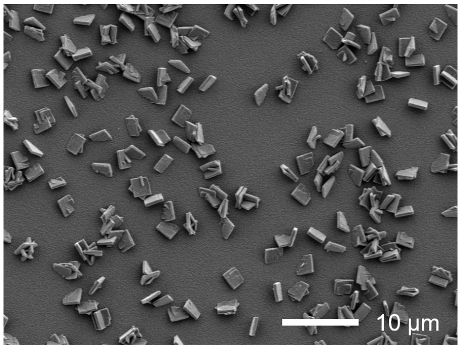

Figure S1. The SEM image of WO_3.

Fig. S1 displays the scanning electron microscope (SEM) image of WO_3, which exhibited morphology of square nanosheet with diameter of ca. 2 μm.

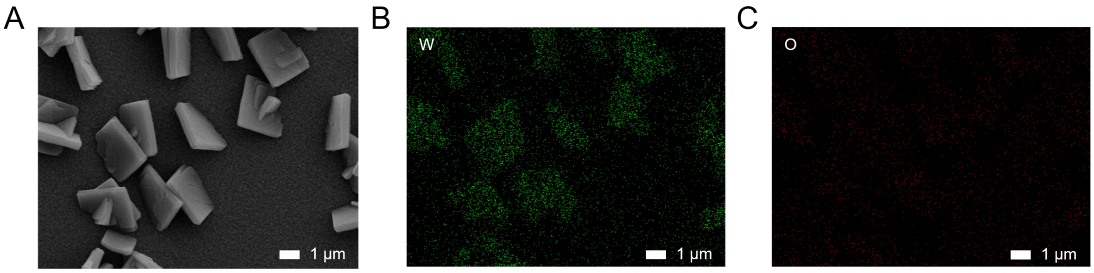

Figure S2. The EDS mapping images of WO_3. (A) The SEM image of WO_3. (B-C) The corresponding EDS mapping of W and O.

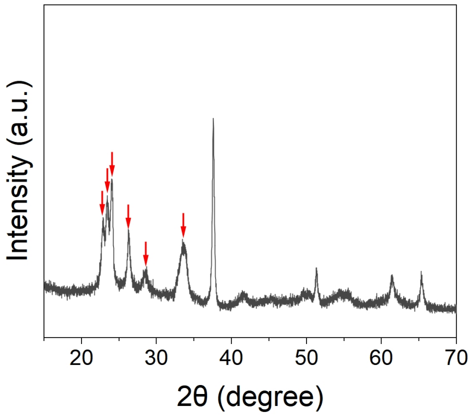

Figure S3. The XRD curve of WO_3/FTO.

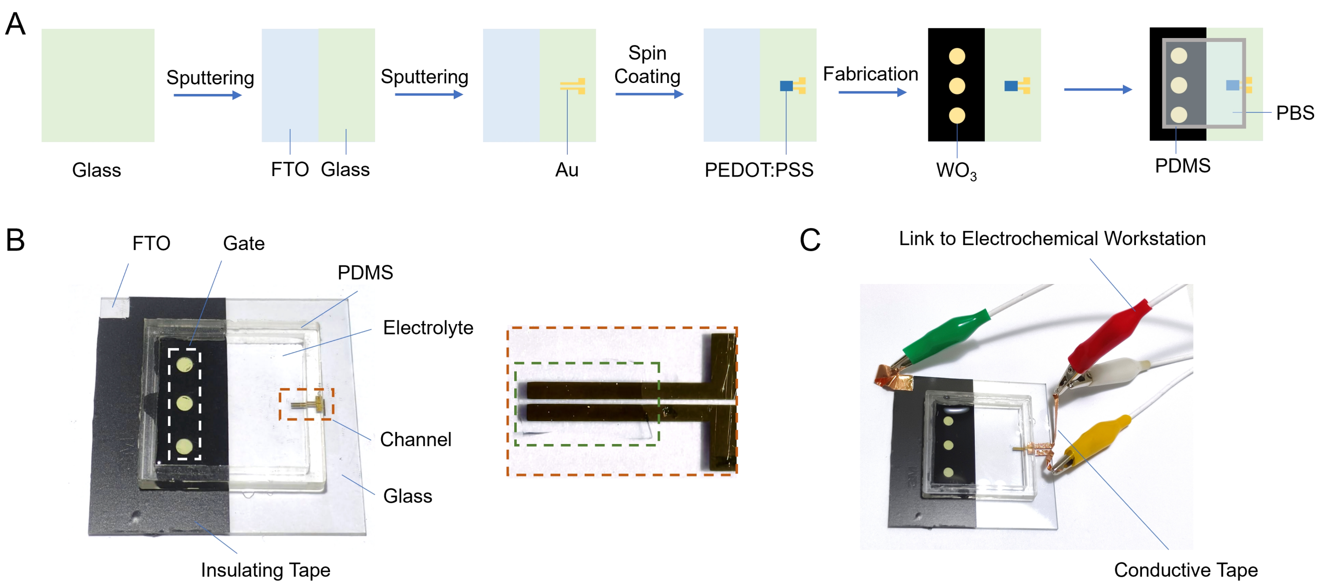

Figure S4. Fabrication step and image of the device.

First, the glass was covered by patterned FTO and gold through magnetron sputtering. Then the PEDOT:PSS channel and the WO_3 gates were fabricated on the substrate. Finally, the electrochemical cell was constructed using the polydimethylsiloxane (PDMS) membrane. For test, the FTO and gold was linked to the electrochemical workstation using the conductive tapes.

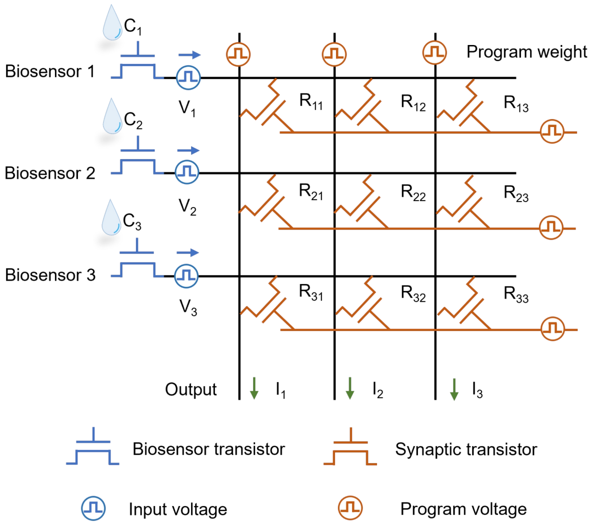

Figure S5. Schematic of the in-biosensing 3×3 neural network.

For the same 3×3 neural network task, a conventional crossbar array requires 3 transistors as biosensors to respectively perceive a biochemical input. The as-produced voltages would be transmitted into a 3×3 interconnected devices as a 3×3 neural network, in which each of the transistor is programmed by a specific voltage to program weight. The complexity and the energy consumption are higher. Such a comparison was added.

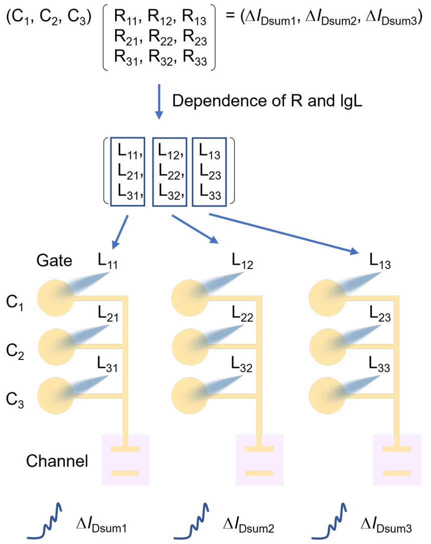

Figure S6. Schematic illustration of the in-sensor VMM process.

To perform the in-sensor VMM, using the linear dependence of R on log(L), the 3×3 R matrix was converted into a 3×3 L matrix, as shown in Fig. S14. Subsequently, to produce a single $\Delta I_{\mathrm{Dsum1}}$, three L values in the first list of the 3×3 L matrix were used to implement corresponding R values. Such process was then repeated for the other two times to use the other six L values in the L matrix and get the other $\Delta I_{\mathrm{Dsum2}}$ and $\Delta I_{\mathrm{Dsum3}}$.

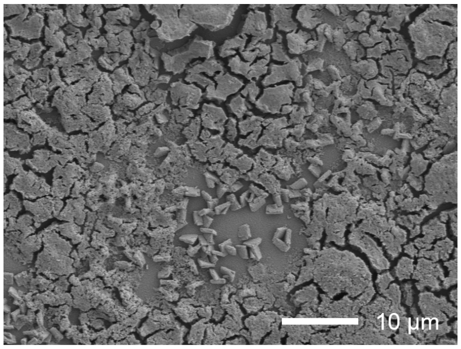

Figure S7. The SEM image of CdS/WO_3.

The CdS appeared as amorphous nanoblocks covered on the WO_3 and the morphology of WO_3 remained unchanged.

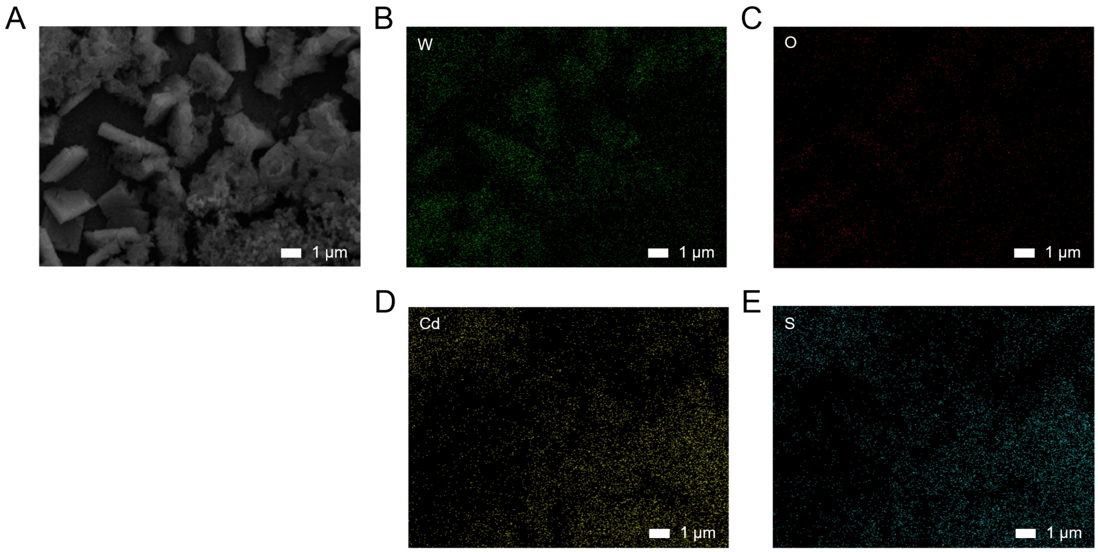

Figure S8. The EDS mapping images of CdS/WO_3. (A) The SEM image of the CdS/WO_3. (B-E) The corresponding EDS mapping of W, O, Cd and S.

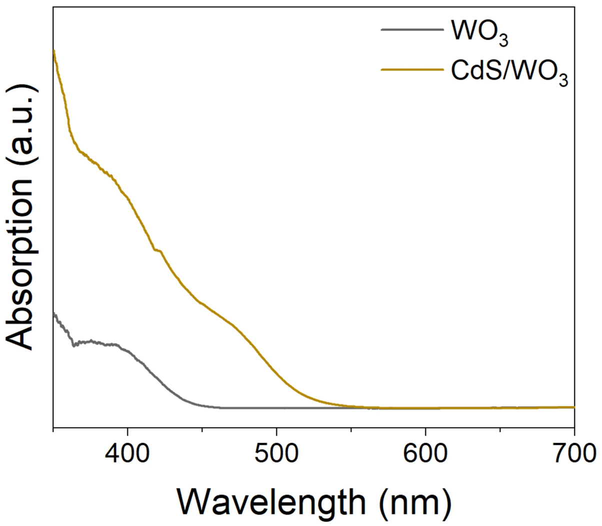

Figure S9. The UV-vis spectra of the CdS/WO_3 and WO_3.

The WO_3 exhibited absorption peak at ca. 400 nm, while the CdS/WO_3 exhibited enhanced absorption with extra peak at ca. 450 nm, which belonged to the characteristic absorption peak of CdS.

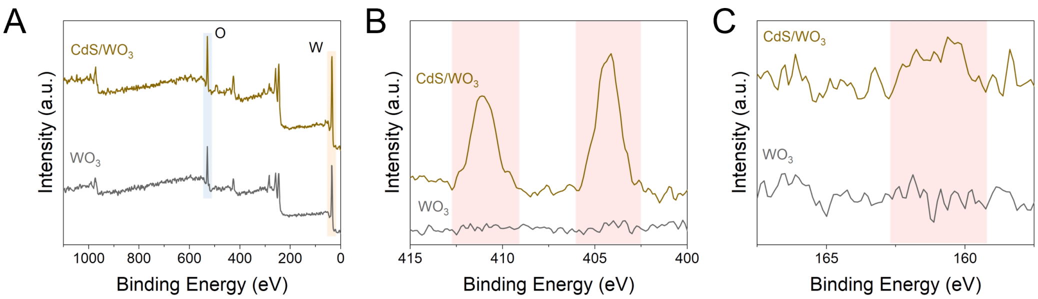

Figure S10. The XPS spectra of CdS/WO_3 and WO_3. (A) Complete XPS spectra of CdS/WO_3 and WO_3. High resolution (B) Cd and (C) S XPS spectra of CdS/WO_3 and WO_3.

As shown, the XPS spectra of the CdS/WO_3 exhibited extra peak of the elements Cd and S compared to that of pristine WO_3, indicating the formation of CdS.

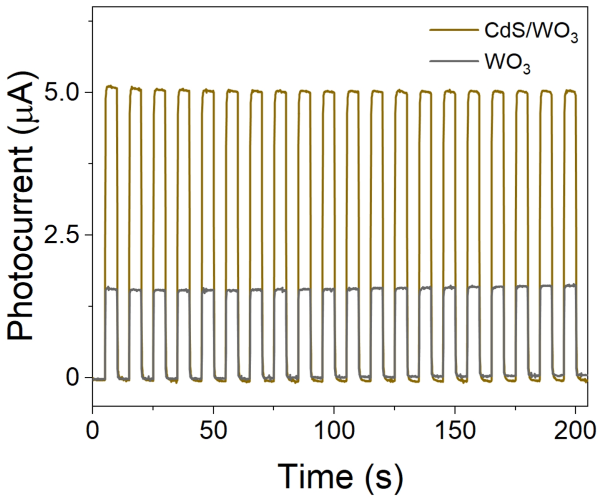

Figure S11. Transient photocurrent responses of CdS/WO_3 and WO_3.

To investigate the photoresponses before and after the treatment of H_2S, the transient photocurrents of CdS/WO_3 and WO_3 were measured using a conventional three-electrode system. Upon identical light illumination, the WO_3 generated an anodic photocurrent of ca. 1.3 μA, while the CdS/WO_3 generated a substantially enhanced photocurrent of ca. 5.0 μA. Both exhibited reproducible photocurrents upon 20 light pulses, indicating the good stability.

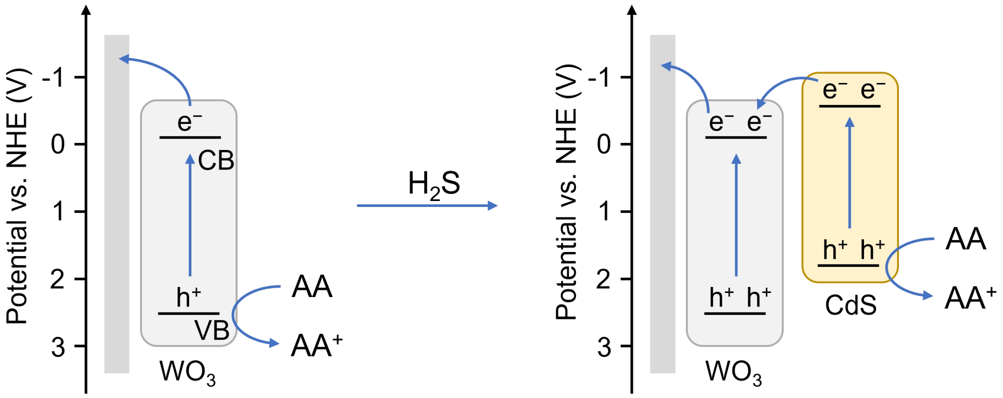

Figure S12. The electron transfer routes upon light illumination.

The enhancement of photoresponse could be attributed to the formation of a type-II heterojunction. For pristine WO_3, upon light illumination, the electrons on the valence band (VB) would be excited to the conductance band (CB) and then transfer to the substrate FTO. After the treatment of H_2S, the formation of type-II heterojunction would boost the electron transfer, thus improving the photocurrent. The ascorbic acid (AA) served as an electron donor in the electrolyte.

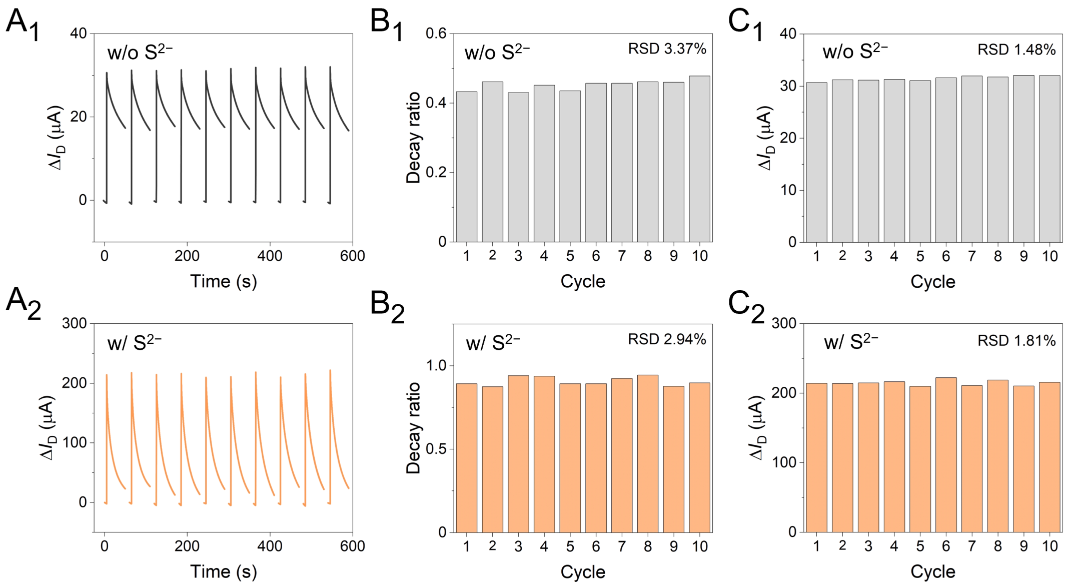

Figure S13. The operation stability of CdS/WO_3 and WO_3.

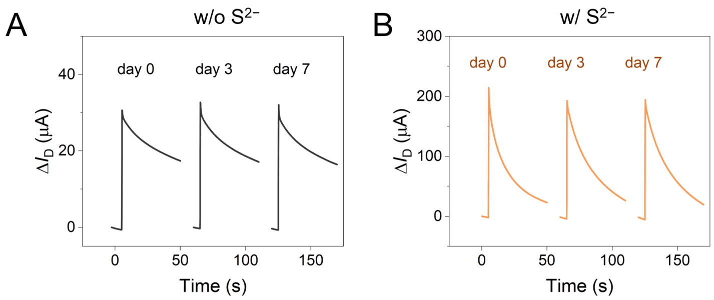

Figure S14. The storage stability of CdS/WO_3 and WO_3.

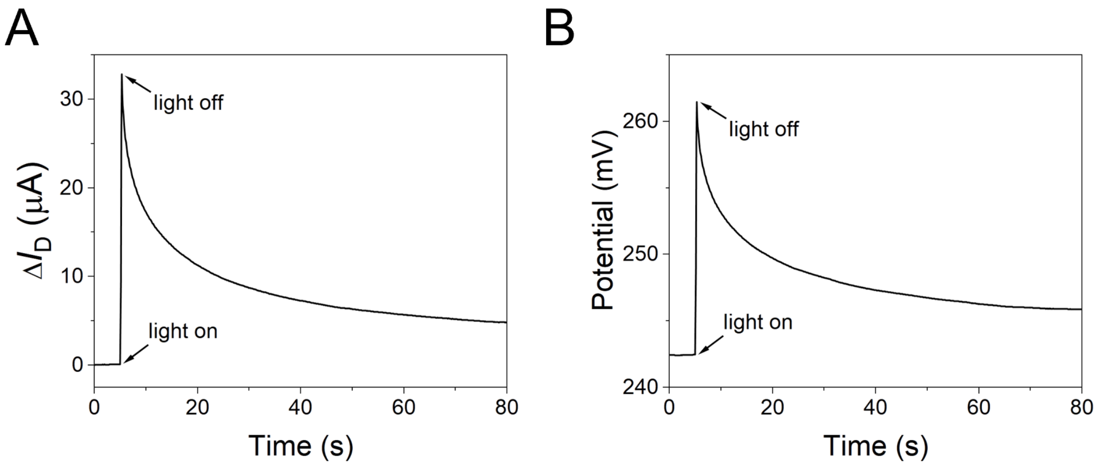

Figure S15. The ΔI_D and OCP upon light pulse. a, The ΔI_D upon light pulse. b, The corresponding OCPT variation.

To investigate the memory behavior, the ΔI_D and open circuit potential (OCP) variation of the device upon a single light pulse were measured. Upon light illumination, the device exhibited ΔI_D and open circuit potential variation. Significantly, after light illumination, both the ΔI_D and open circuit potential exhibited slow retention and could not return to the initial level after 75 s, demonstrating the memory property.

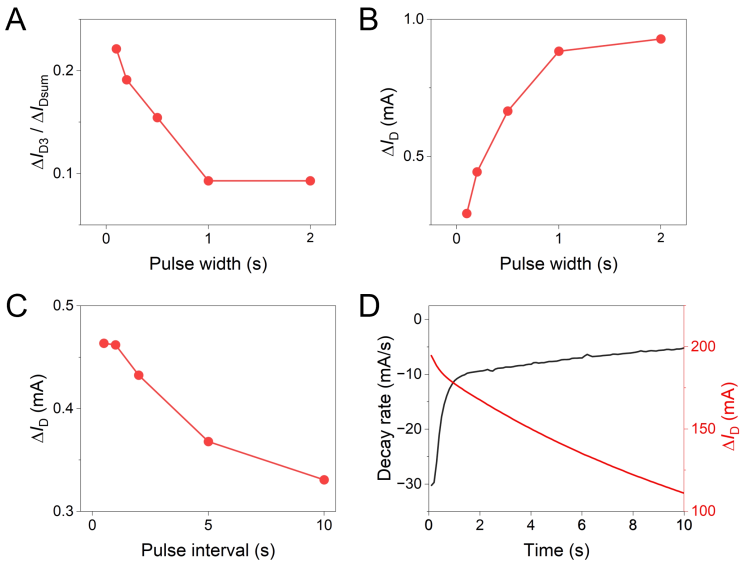

Figure S16. Optimization of the pulse width and interval.

As shown in Fig. S16A, increased pulse width would lead lower $\Delta I_{D3}/\Delta I_{Dsum}$, indicating the increased nonlinearity of the accumulation, which would influence the precision of the VMM. However, as shown in Fig. S16B, small pulse width resulted in low ΔI_D, which would reduce the sensitivity of biosensing. Considering both of them, 0.2 s was selected as the pulse width.

On the other hand, to ensure the correction of VMM, the readout time was set as (pulse width+ pulse interval)×3+quiet time. As shown in Fig. S16C, increased pulse interval would lead to decreased ΔI_{Dsum}, which would reduce the sensitivity of biosensing. However, increased pulse interval would lead to increased readout time. As shown in Fig. S16D, the decay of ΔI_D would be too fast when the readout time is lower than c.a. 1.5 s, which would influence the stability of recording the result. Considering both of them, 2 s was selected as the pulse interval.

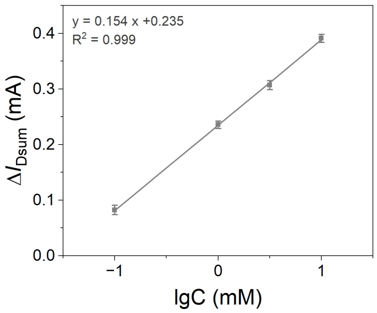

Figure S17. TheΔI_{Dsum} responses with the treatment of H_2S with variable concentrations.

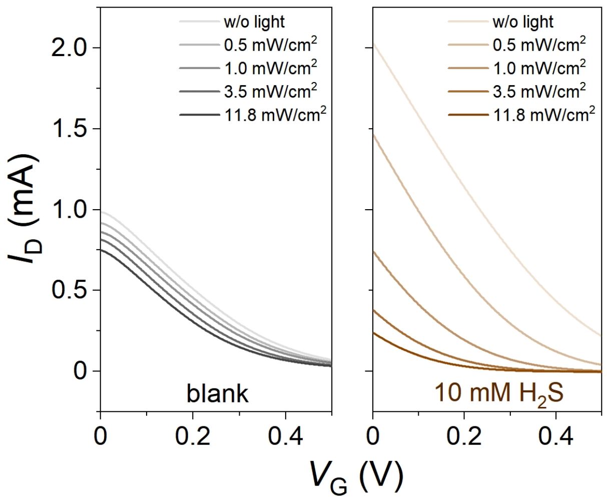

Figure S18. The transfer characteristic curves before and after the treatment of H_2S upon light illumination with different intensities.

Before the treatment of H_2S, the transfer characteristic curves exhibited slight leftwards shift with increased light intensity. After the treatment of H_2S, the transfer characteristic curves exhibited more substantial leftwards shift upon same light illumination, indicating the light-tunable sensitivity to H_2S.

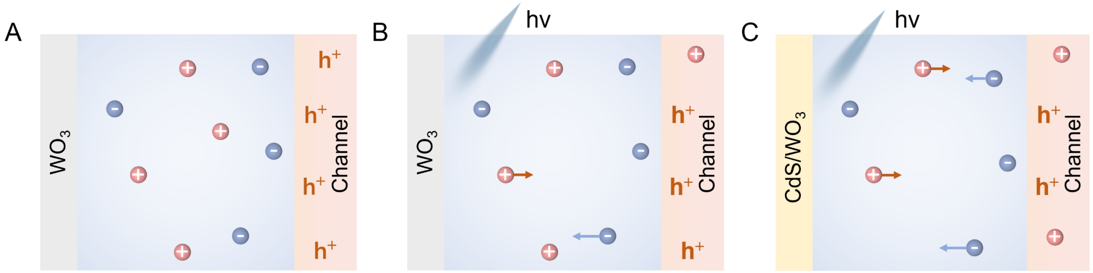

Figure S19. The ion migration of WO_3 and CdS/WO_3 upon light illumination.

The H_2S-induced enhancement of ΔI_D could be illustrated from the perspective of ion migration. Upon light illumination, the photo-induced voltage on the gate would drive cations into the channel, leading to the variation of channel conductivity. After the treatment of H_2S, the improved photoresponse would drive more cations into the channel, leading to higher ΔI_D response.

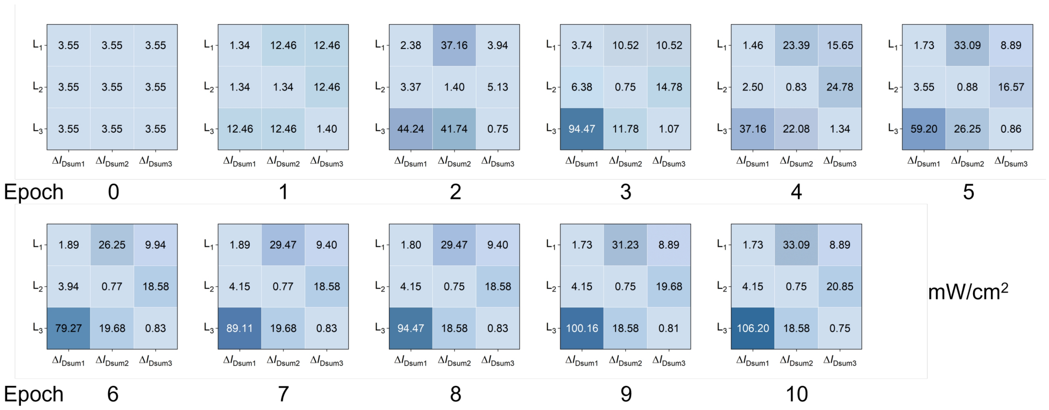

Figure S20. The predicted variation of synaptic weight matrix.

With increased training epochs, three groups of L_1-L_3 for generating ΔI_{Dsum1}-ΔI_{Dsum3} gradually changed. Specifically, for ΔI_{Dsum1}, the L_1 slightly decreased and L_3 enhanced. For ΔI_{Dsum2}, the L_1 and L_3 enhanced while the L_2 decreased. For ΔI_{Dsum3}, the L_1 and L_2 enhanced while the L_3 decreased. Such results were consistent with the characteristics of three samples.

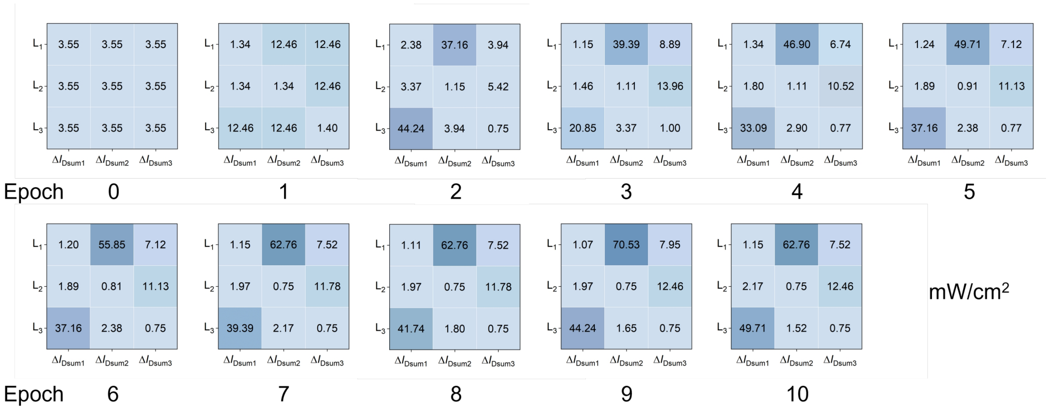

Figure S21. The variation of synaptic weight matrix during *in-situ* training.

After *in-situ* training, the synaptic weight matrix exhibited variation similar with the predicted ones. The results were also consistent with the characteristics of the three samples.

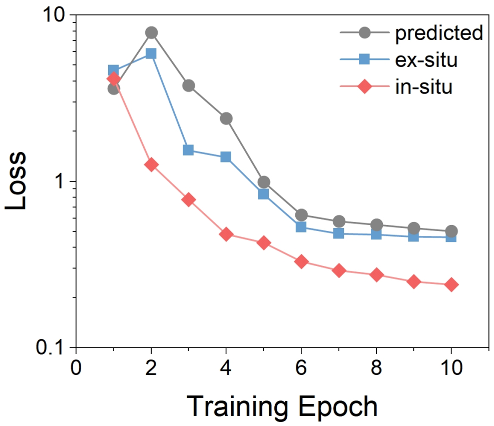

Figure S22. The variation of Loss during training.

The Loss values gradually decreased with increased epochs. The *ex-situ* training exhibited similar decrease speed with the predicted training, which was due to their identical synaptic weight matrix. Notably, the Loss value of the *in-situ* training decreased most rapidly, indicating its superior classification capability.

Note S1

According to the principle of ANN, the theoretical ΔI_{Dsum} could be calculated as:

$$\Delta I_{Dsum1} = R_{11}lgC_1 + R_{21}lgC_2 + R_{31}lgC_3$$

$$\Delta I_{Dsum2} = R_{12}lgC_1 + R_{22}lgC_2 + R_{32}lgC_3$$

$$\Delta I_{Dsum3} = R_{13}lgC_1 + R_{23}lgC_2 + R_{33}lgC_3$$

The functions of the three decision surfaces could be calculated by letting $\Delta I_{Dsum1} = \Delta I_{Dsum2}$, $\Delta I_{Dsum2} = \Delta I_{Dsum3}$, and $\Delta I_{Dsum1} = \Delta I_{Dsum3}$. On the other hand, the coordinates of the microRNA fingerprints were described as (ΔI_{D1}, ΔI_{D2}, ΔI_{D3}), in which ΔI_{D1}-ΔI_{D3} were the ΔI_D responses upon a light pulse on the photogate corresponding to C_1, C_2 and C_3, respectively. The functions of decision surfaces and coordinates of fingerprints were then normalized.

Note that the three decision surfaces intersected at a single line (0.2x=0.28y=0.29z). The function of the normal plane could be calculated. Afterwards, the decision surfaces and the fingerprints were projected into the normal plane

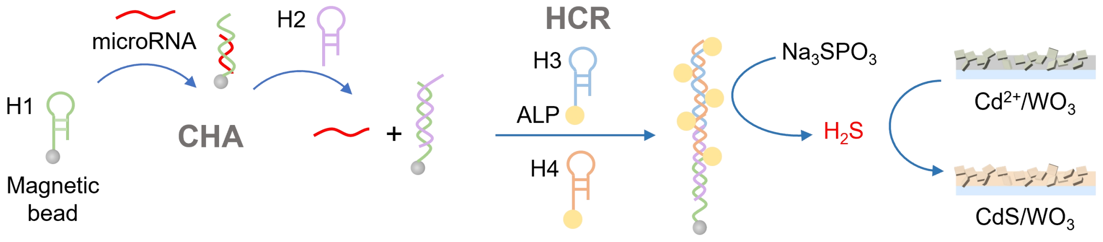

Figure S23. Nucleic acid strategy for microRNA biosensing.

MicroRNA-triggered catalytic hairpin assembly (CHA) and hybridization chain reaction (HCR) were combined with alkaline phosphatase (ALP)-catalyzed generation of H_2S, the subsequent production of CdS onto the three photogates permitted them to sense different microRNA, i.e. microRNA-21, microRNA-221 and microRNA-133a, respectively.

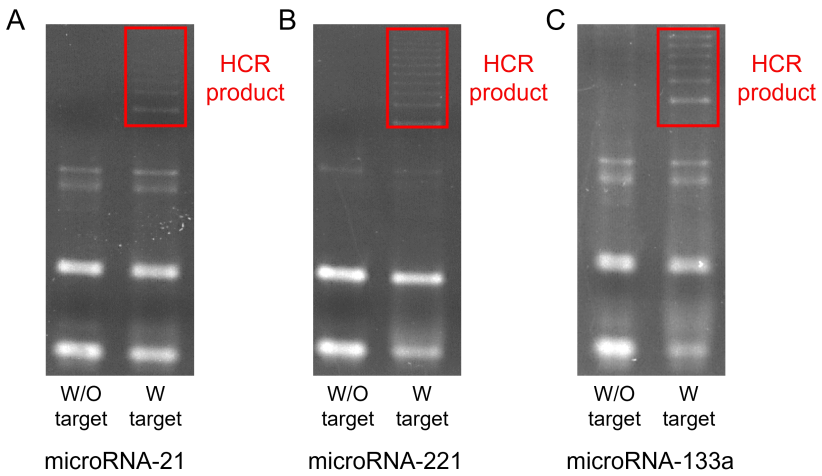

Figure S24. PAGE analysis of nucleic acid strategy.

The HCR products could be generated only when the target microRNA existed, indicating the feasibility of the as-designed nucleic acid strategy.

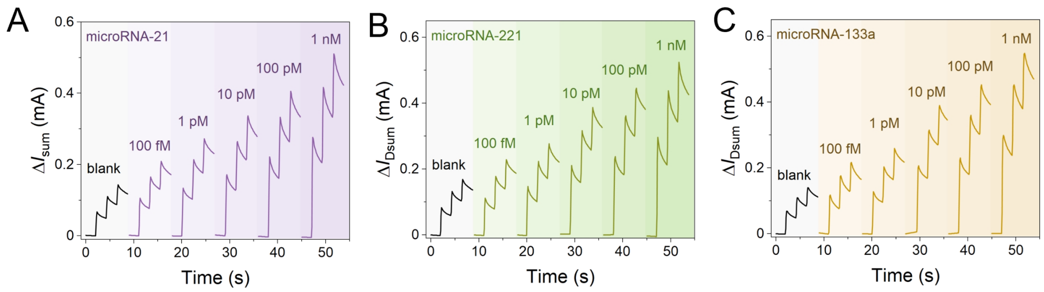

Figure S25. Reponses before and after the treatment of microRNA.

Upon three light pulses on the individual photogate, the ΔI_{Dsum} gradually increased with the increased concentration of corresponding target microRNA, indicating the capability for microRNA biosensing.

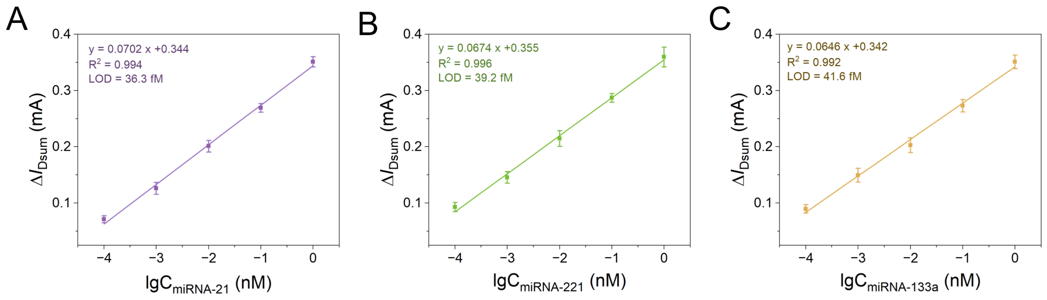

Figure S26. The calibration curves of microRNA detection.

The ΔI_{Dsum} and logarithm of microRNA concentrations exhibited a good linear relationship, which supported the multiplication operation. The limit of detection (LOD) (S>3N) of microRNA-21, microRNA-221 and microRNA-133a were 36.3, 39.2 and 41.6 fM, respectively.

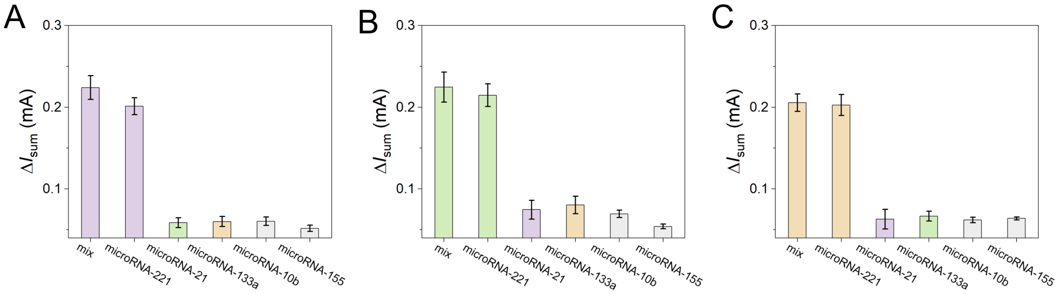

Figure S27. The selectivity of microRNA detection. The responses with 10 pM (A) microRNA-21, (B) microRNA-221 and (C) microRNA-133a against 100 pM interferences.

In the presence of tenfold higher concentration of interferences, only the target RNA and its mixture can produce significantly enhanced ΔI_{Dsum}, indicating the good selectivity.

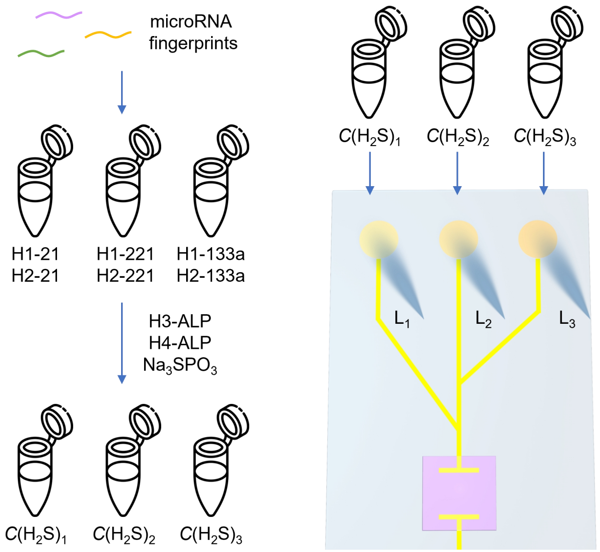

Figure S28. The process of microRNA fingerprint sensing.

The microRNA fingerprint sensing process was illustrated. As shown, the microRNA fingerprints sample was added into three tubes with specific H1 and H2, which were used for microRNA-21, microRNA-221 and microRNA-133a detection, respectively. H3-ALP, H4-ALP and Na_3SPO_3 were then added to produce H_2S, whose concentrations were dependent on the concentrations of the target microRNA. The three tubes of H_2S were then reacted with corresponding Cd^{2+}/WO_3 photogates and L_1-L_3 were applied for classification.

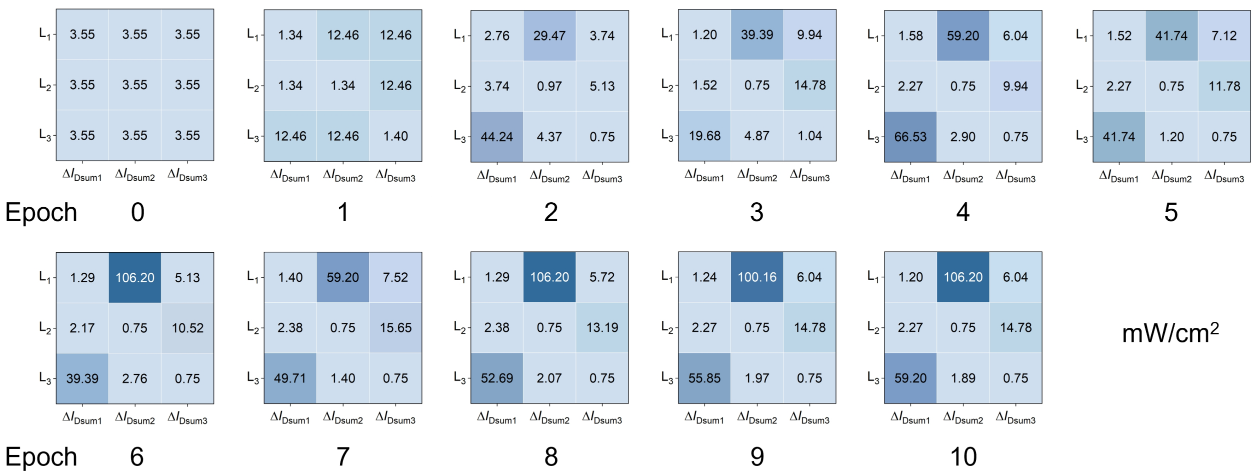

Figure S29. The predicted variation of synaptic weight matrix towards classification of real biological samples.

With increased training epochs, for ΔI_{Dsum1}, the L_1 slightly decreased and L_3 enhanced. For ΔI_{Dsum2}, the L_1 enhanced while the L_2 decreased. For ΔI_{Dsum3}, the L_2 enhanced while the L_3 decreased. Such results were consistent with the characteristics of three microRNA fingerprints, indicating the successful training.

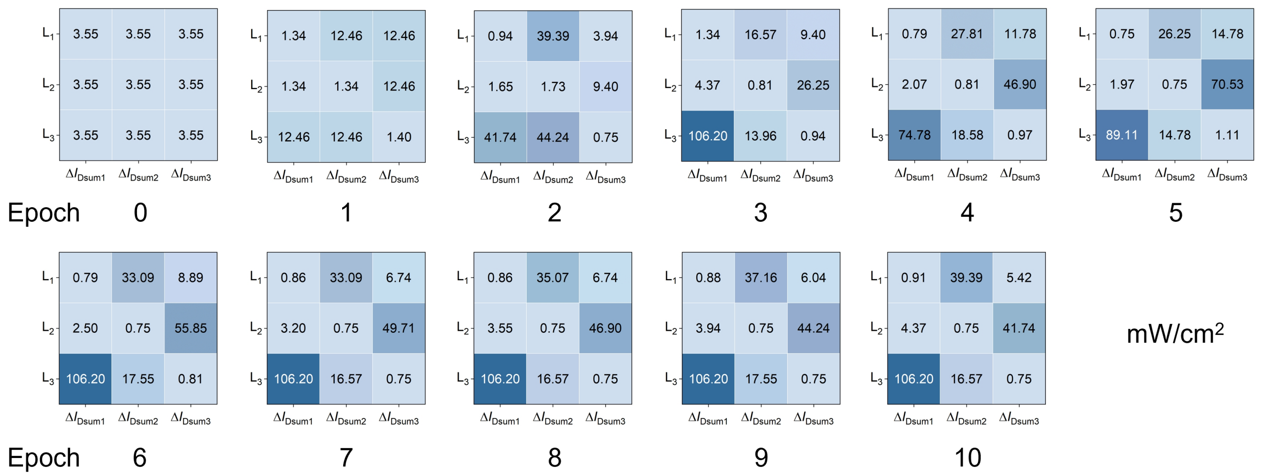

Figure S30. The variation of synaptic weight matrix during *in-situ* training towards classification of real biological samples.

The synaptic weight matrix exhibited similar variation with the predicted one, indicating the successful training.

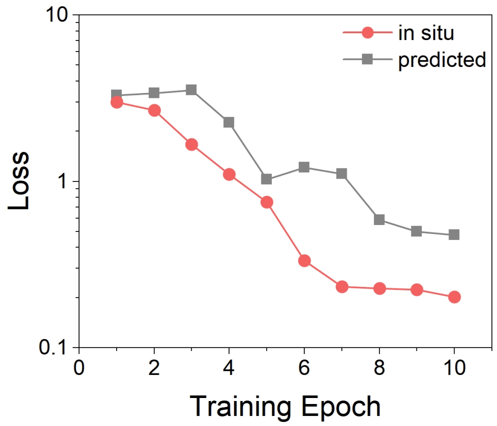

Figure S31. The variation of Loss during training towards classification of real biological samples.

Both the Loss values of the predicted and *in-situ* training gradually decreased with increased training epoch. Significantly, the Loss values of the *in-situ* training exhibited faster decrease speed, indicating its superior classification capability.

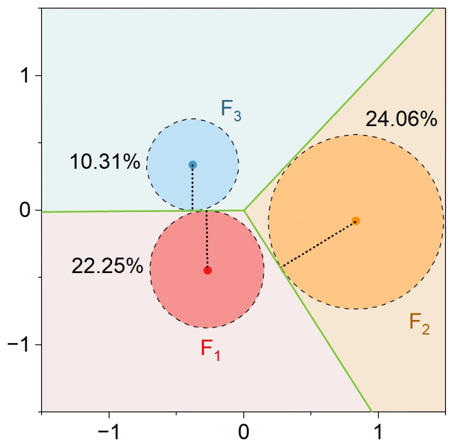

Figure S32. The projection of the decision surfaces and fingerprints of real biological samples on the normal plane.

To theoretically study the robustness, the decision surfaces and fingerprints were projected onto the normal plane according to the aforementioned model in Fig. 3A. As shown, the whole space was separated into three parts and the three fingerprints were correspondingly located in each one of them. As calculated, it could tolerate maximum relative mean square deviations of 22.25%, 24.06% and 10.31% for 100% accurate classification of F_1, F_2 and F_3, respectively, indicating the good robustness.

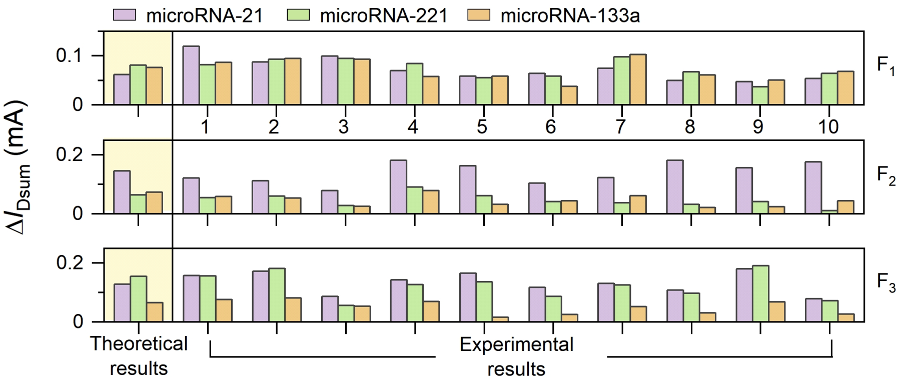

Figure S33. The ΔI_{Dsum} upon three light pulses on each individual photogate of 30 random samples.

To study the robustness of the classification of microRNA fingerprints in real biological samples, 30 random cell lysates were used for testing. As shown, upon three light pulses on each individual photogate, the devices generated randomly enhanced or decreased ΔI_{Dsum} compared with the theoretical results, which was due to the differences between the samples and random error.

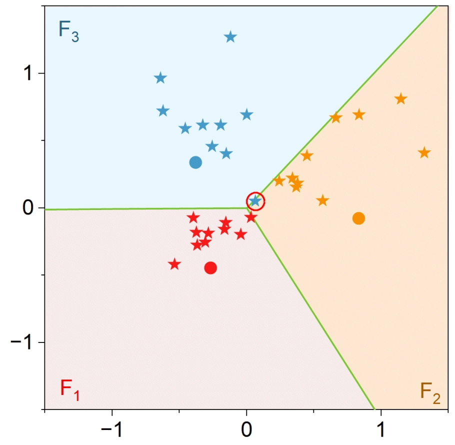

Figure S34. The projection of the corresponding ΔI_{Dsum} on the normal plane.

For theoretical prediction of the classification results, all the ΔI_{Dsum} were projected into the normal plane. As shown, most of the points were correctly classified, with only 1 mistake, i.e., one blue point belonging to the F_3 was separated into the orange F_2 part. Such result indicated the good classification capability against the interference from device-to-device variation.

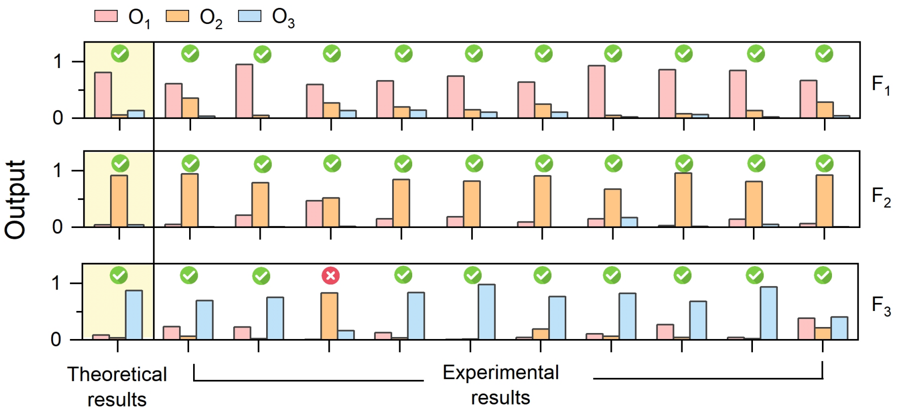

Figure S35. The outputs of classifying 30 samples.

For further experimental validation, the outputs of the 30 classifications were obtained. As shown, only one mistake was made, i.e., one sample belonging to the F_3 was incorrectly classified as F_2, which was consistent with the prediction, confirming the accurate classification.

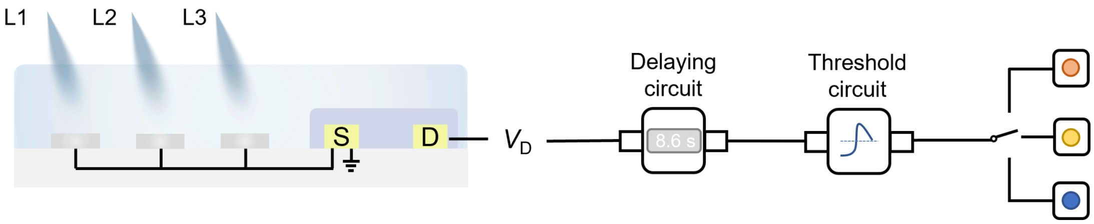

Figure S36. Schematic illustration of controlling LED lights.

To rapidly present the classification results, the transistor was then linked with a threshold circuit and a delay circuit to control the LED lights. As shown, after delaying 8.6 s for generating ΔI_{Dsum}, the I_D were compared with the as-set threshold to determine which light should be turned on. The highest ΔI_{Dsum1}-ΔI_{Dsum3} would respectively result in the turning on of the red, orange and blue light.

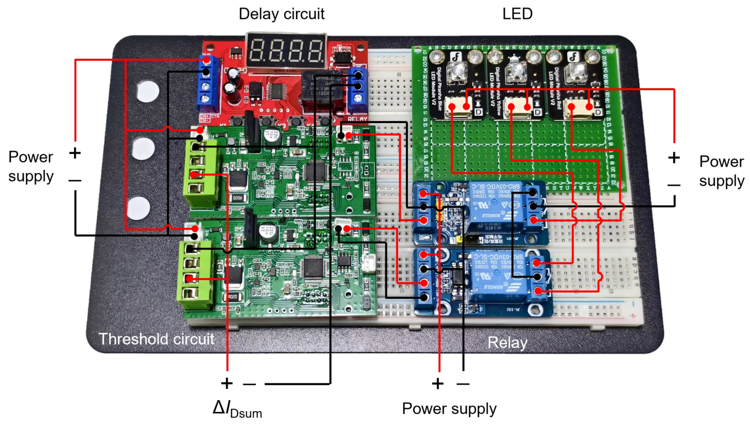

Figure S37. Image of the delay and the threshold circuits.

The delay circuit would open the threshold circuit at 8.6 s. The threshold circuit would initially judge whether the blue LED should be lighted on, then the orange one and then the red one.

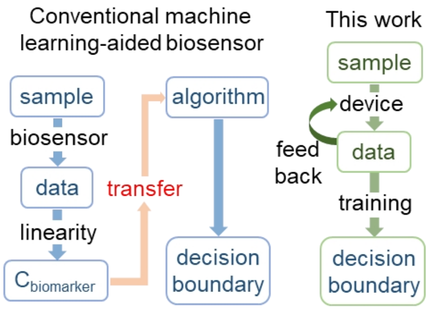

Figure S38. Schematic illustration of decision boundary acquisition using conventional method and in-biosensing ANN.

Note S2

The decision boundaries could be described as :

$$(R_{11}- R_{12})\lg C_1 + (R_{21}- R_{22})\lg C_2 + (R_{31}- R_{32})\lg C_3 = 0$$

$$(R_{13}- R_{12})\lg C_1 + (R_{23}- R_{22})\lg C_2 + (R_{33}- R_{32})\lg C_3 = 0$$

$$(R_{13}- R_{11})\lg C_1 + (R_{23}- R_{21})\lg C_2 + (R_{33}- R_{31})\lg C_3 = 0$$

In Fig. 4E, by using the first function and letting $\lg C_2$ equal to the average value of F_1 and F_2, the functions of this method and conventional ANN method could be calculated as:

$$\lg C_1 = 0.66\lg C_3 - 1.05$$

$$\lg C_1 = 0.74\lg C_3 - 0.63$$

In Fig. 4F, by using the second function and letting $\lg C_1$ equal to the average value of F_2 and F_3, the functions of this method and conventional ANN method could be calculated as:

$$\lg C_2 = 0.93\lg C_3 - 0.54$$

$$\lg C_2 = 0.40\lg C_3 - 2.00$$

In Fig. 4G, by using the three functions and letting $\lg C_3$ equal to the average value of F_1, F_2 and F_3, the functions of this method and conventional ANN method could be calculated as:

$$\lg C_2 = -0.54\lg C_1 - 7.54$$

$$\lg C_2 = 1.59\lg C_1 + 2.06$$

$$\lg C_2 = 0.41 \lg C_1 - 2.77$$

$$\lg C_2 = -0.97 \lg C_1 - 7.40$$

$$\lg C_2 = 1.49\lg C_1 + 1.41$$

$$\lg C_2 = 0.58\lg C_1 - 1.77$$

Note that the boundaries of this method and conventional ANN exhibited similar functions, indicating the feasibility of this method to produce the decision boundaries.

.

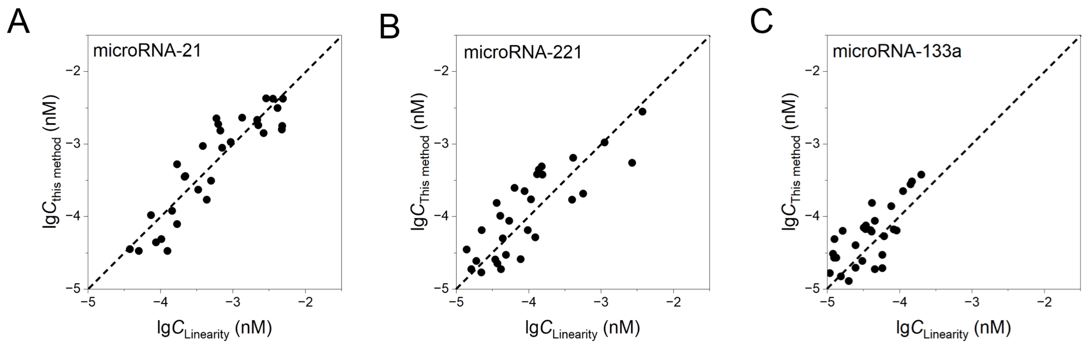

Figure S39. The comparation of microRNA concentrations calculated by this method and linearity functions.

The specific concentrations of three microRNA were respectively calculated by this method and the linearity functions using the data from real samples F_1-F_3. As for this method, the concentrations of three microRNA were calculated by dividing the ΔI_{Dsum} with the R matrix. As shown, the concentrations calculated by this method and the linearity functions exhibited similar values, indicating the capability to obtain the specific concentrations.

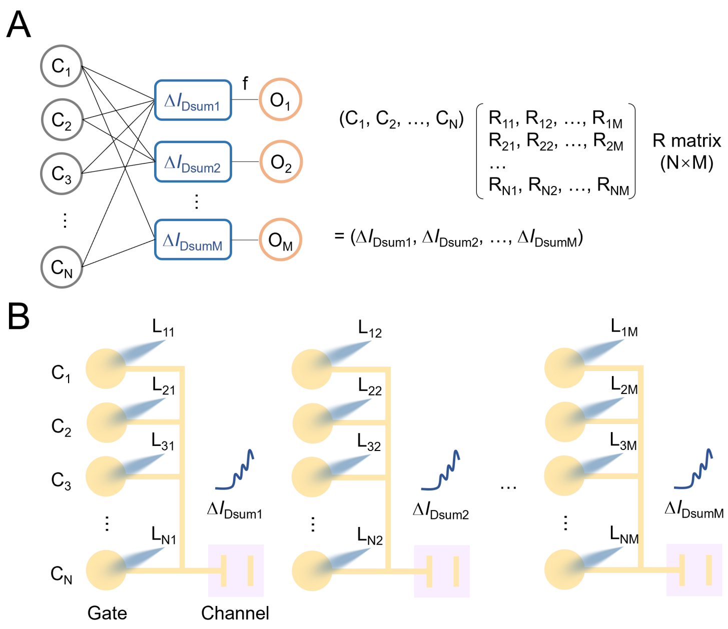

Figure S40. Schematic of an in-sensor N×M ANN.

A neural network with N inputs and M outputs requires the VMM between a (C_1, C_2,⋯, C_N) and a N×M R matrix to obtain the output of (ΔI_{Dsum1}, ΔI_{Dsum2},⋯, ΔI_{DsumN}). Specifically, it needs an OPECT device with N photogates to respectively perceive a specific biochemical input. Upon N subsequent light pulses with individual intensities (L_{11}, L_{21},⋯, L_{N1}), the device can produce a ΔI_{Dsum}. Such process is repeated for M times using the N×M L matrix to perform the complete VMM and thus obtain the output.

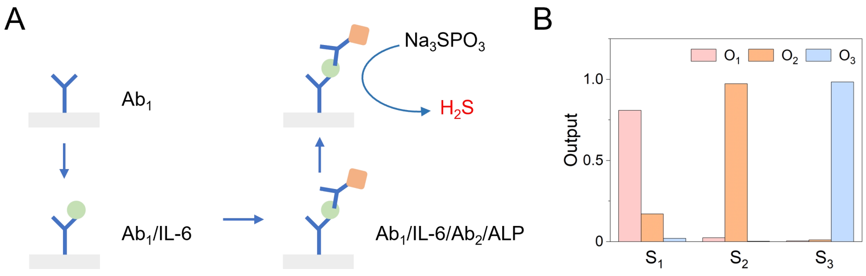

Figure S41. The in-sensor ANN with the input of IL-6.

To study the feasibility of this strategy to other target, interleukin-6 (IL-6) was chosen as a model target using the classic enzyme linked immunosorbent assay. As shown in Fig. S41A, the IL-6 would be captured by the ALP-labeled antibody (Ab), which could subsequently catalyze the production of S^{2-}. The as-produced S^{2-} could be detected by our electrode. We explore a typical linear separation task with concentration combinations of IL-6 as the model samples (denoted as S_1 = (1 pg/mL, 1 pg/mL, 1 pg/mL), S_2 = (1 ng/mL, 1 pg/mL, 1 pg/mL) and S_3 = (1 ng/mL, 1 ng/mL, 1 pg/mL)). As shown in Fig. S41B, the output showed that all the three samples could be well divided.

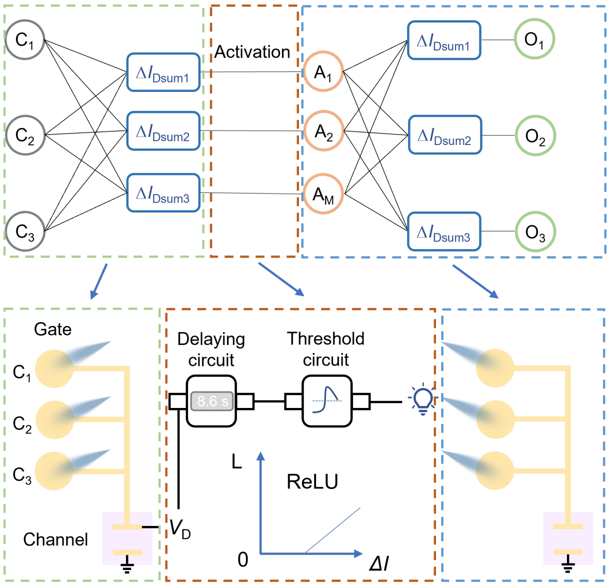

Figure S42. Schematic illustration of the in-biosensing multi-layer ANN.

For multi-layer ANN, the activation function could be performed by using the delay circuit and threshold circuit to execute the ReLU function as activation, in which the ΔI_{Dsum} is detected to control the light illumination on the second device. The second device then works as hidden layer, on which the second VMM is applied.

Table S1. DNA sequences used for microRNA detection

Name	Sequences (5'-3')
**microRNA-21**	UAGCUUAUCAGACUGAUGUUGA
**microRNA-221**	AGCUACAUUGUCUGCUGGGUUUC
**microRNA-133a**	AGCUGGUAAAAUGGAACCAAAU
**H1-21**	TCAACATCAGTCTGATAAGCTACTACAACTGGCGGGGTAGCTTATCAGACT
**H2-21**	CTACAACTGGCGGGGAAGCTACCCCGCCAGTTGTAGTAGCTTATCAGACT
**H1-221**	GAAACCCAGCAGACAATGTAGCTCTACAACTGGCGGGGAGCTACATTGTCTG
**H2-221**	CTACAACTGGCGGGGGTAGCTCCCCGCCAGTTGTAGAGCTACATTGTCTG
**H1-133a**	ATTTGGTTCCATTTTACCAGCTCTACAACTGGCGGGGAGCTGGTAAAATGG
**H2-133a**	CTACAACTGGCGGGGCCAGCTCCCCGCCAGTTGTAGAGCTGGTAAAATGG
**H3-ALP**	ALP-CTGGCGGGGAGGAAGCCCCGCCAGTTGTAG
**H4-ALP**	CTTCCTCCCCGCCAGCTACAACTGGCGGGG-ALP
